# Experimental Study of Carbonation and Chloride Resistance of Self-Compacting Concretes with a High Content of Fly Ash and Metakaolin, with and Without Hydrated Lime

**DOI:** 10.3390/ma18020422

**Published:** 2025-01-17

**Authors:** Marcos Alyssandro S. dos Anjos, Aires Camões, Raphaele Malheiro, Cinthia Maia Pederneiras, Lorena K. S. Peixoto

**Affiliations:** 1 Department of Civil Engineering, Federal Institute of Education, Science and Technology of Paraíba (IFPB), João Pessoa 58015-435, Brazil; 2 The Centre for Territory, Environment and Construction (CTAC), Department of Civil Engineering, University of Minho, Campus de Azurém, 4800-058 Guimarães, Portugal; aires@civil.uminho.pt (A.C.); raphaelemalheiro@civil.uminho.pt (R.M.); 3 c5Lab Sustainable Construction Materials Association, 2795-242 Lisbon, Portugal; cpederneiras@c5lab.pt; 4 Federal Institute of Education, Science and Technology of Rio Grande do Norte (IFRN), São Gonçalo do Amarante 59291-727, Brazil; lorena.peixoto@ifrn.edu.br

**Keywords:** self-compacting concrete, low cement content, accelerated carbonation, diffusion of chloride ions, resistivity

## Abstract

The durability of reinforced concrete is associated with several factors that can trigger the corrosion of reinforcement bars. Among these factors, the most significant are chloride-ion attack and carbonation. This study evaluated, through accelerated testing, self-compacting concretes (SCCs) with reduced cement content in binary, ternary, and quaternary mixtures using high-early-strength Portland cement, fly ash (FA), metakaolin (MK), and hydrated lime (HL). These systems are proposed to address the slow compressive strength gains at 28 days in concretes with high fly ash content and to minimise the effects of carbonation in concretes with high levels of mineral additives. Laboratory tests were conducted to measure chloride-ion migration in a non-steady-state system, accelerated carbonation in a controlled chamber, electrical resistivity, void indices, and compressive strength. Based on the results obtained, it was found that the combined use of MK, FA, and HL was effective in reducing cement consumption to extreme levels, such as 120 and 150 kg/m^3^, while still achieving durability indices superior to those of SCCs with cement consumption of 500 kg/m^3^.

## 1. Introduction

Self-compacting concretes (SCCs) have many fields of application, but many times, they are associated with high cement contents (>300 kg/m^3^) to achieve the desired properties [1,2,3,4]. Reduced-cement-content self-compacting concretes using supplementary cement materials have benefits related to the economy, energy efficiency, and greenhouse gas emissions. Nevertheless, these materials sometimes still have a cement consumption of more than 300 kg/m^3^ [5,6,7,8].

Self-compacting concretes with a high volume of fly ash (≥50% of total cementitious material) have been studied, reaching satisfactory results related to strength [9,10,11,12,13,14]. However, it is important to consider two important issues when a high volume of fly ash is used: the low compressive strength during the early stages and carbonation performance. Concerning the first issue, a slower increase in strength is associated with these high-volume fly ash concretes (HVFACs) [14,15,16,17], although, ultimately, these materials become stronger with age [17,18,19,20].

The lower compressive strength gain during the early stage is related to the production of less calcium hydroxide by hydration, either due to the smaller amount of cement or consumption by the fly ash. This behaviour can also be related to the pH of the pore solution (less alkaline) which reduces the solubility of the amorphous silica and alumina of the pozzolan, delaying the early activation of fly ash [14,21,22,23]. Different approaches have been used to improve the early strength of HVFACs. They include the use of high-fineness fly ash, the addition of super-pozzolanic materials like silica fume, metakaolin, and rice husk ash, nano-modifying the self-compacting concrete with a high volume of fly ash, and the addition of hydrated lime [24,25,26,27,28,29].

Although promising results have been achieved in terms of strength, the durability approach should be assessed. On one hand, some studies show that less cement consumption associated with the presence of less portlandite can compromise the durability behaviour, mainly in terms of carbonation (the second important issue), which consumes portlandite [30,31,32,33,34]. On the other hand, high-volume fly ash SCC had significantly lower weight losses during the acid attack, as well as reduced chloride-ion diffusion, when compared to ordinary concretes with the same strength grades [15,35]. Furthermore, the absorption, sorptivity, and permeable voids (apparent volume) decrease with an increase in fly ash contents and the curing period for SCC, with high-volume fly ash content being substantial at higher ages (28 to 56 days) [36]. Concretes containing high-volume fly ash and hydrated lime presented an inferior accumulated charge density and coefficient of chloride diffusion than the concrete with high-early-strength cement used as a reference [21]. The kinetics of the cement’s hydration and pozzolanic activity can be impacted by the presence of hydrated lime, which also affects carbonation [30,31].

The lime addition ensures better and complete hydration of fly ash in systems with low cement content in the mixture, improving durability and strength [14,31,37]. Determining the necessary amount of added hydrated lime to provide a minimum amount of free lime is a challenge, as it depends not only on the amount of cement and fly ash but also on the fineness of fly ash and other super-pozzolanas. Sanjuán et al. [38,39] studied ternary blended cements that incorporated limestone of varying fineness. The addition of lime affects the cement’s properties through particle size distribution and content. The effects observed include dilution, filler, nucleation, and chemical interactions.

In addition, the hydrated lime addition has also been proposed to enhance the self-healing capability of high-volume fly ash-incorporated cementitious composites [14,36,37,40,41]. Srinivas et al. [42] also investigated the addition of limestone in blended cement, with the authors concluding that this incorporation led to an increase in compressive strength.

This paper contributes to this scientific discussion, carrying out experiments on the durability properties of reduced-cement-content self-compacting concrete with low cement content using a high volume of fly ash and metakaolin, with and without hydrated lime. This research studies the durability of concretes whose mechanical performance has already been studied by Anjos et al. [43,44]. The present work evaluates accelerated carbonation, the presence of aluminium oxide and calcium oxides in the mixtures, chloride-ion diffusion, electrical resistivity, and air voids in SCC with low cement contents. The main weaknesses and potential of self-compacting concrete with cement consumption between 120 kg/m^3^ and 200 kg/m^3^ are highlighted, with a focus on durability (considering the risk of corrosion), which is part of the innovative nature of this research work.

## 2. Materials and Methods

### 2.1. Materials

**Figure 1:**
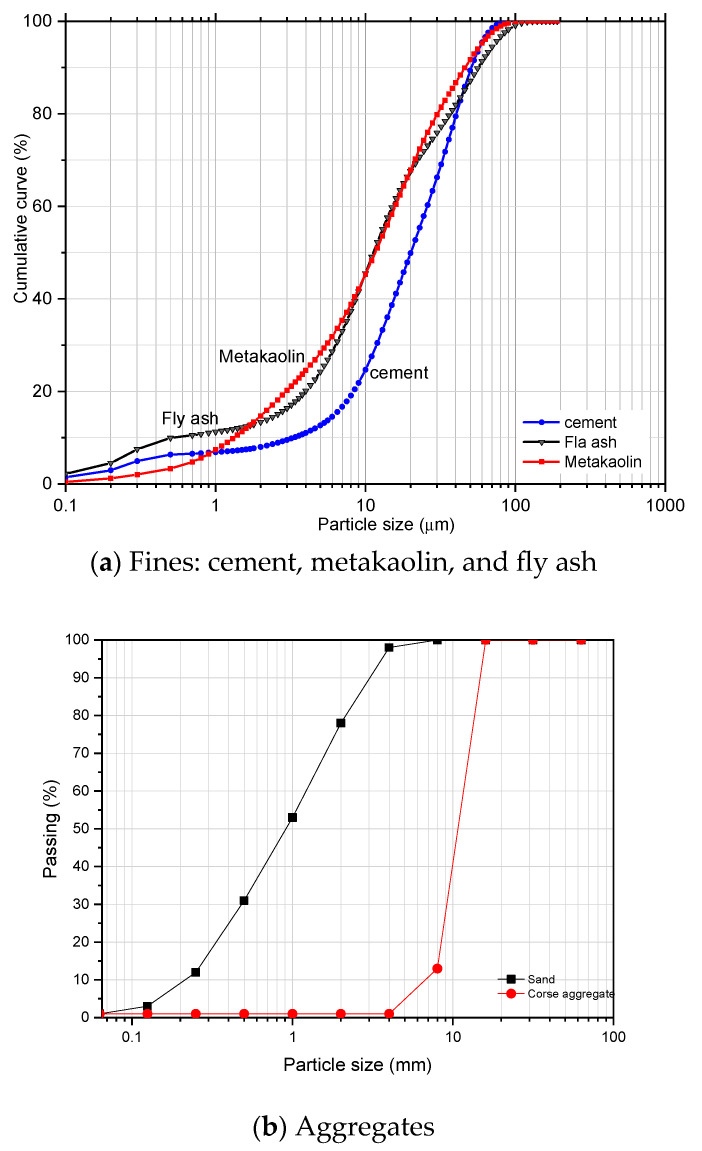
Particle size distribution.

Portland cement CEM I 42.5R (C), Class B fly ash (FA) complying with EN 450-1 [45], metakaolin (MK), and hydrated lime (HL) commercials were used as binders. We also used a coarse crushed aggregate from a granite quarry (G) (Dmax = 16 mm), a fine natural river aggregate (S) (Dmax = 4 mm), tap water (W), and a polycarboxylate-based superplasticiser (SP). The particle size distributions of the materials are shown in Figure 1. More details about the chemical analysis and particle size distribution curves of cement, hydrated lime, metakaolin, fly ash, and aggregates used in this research can be seen in the studies by Anjos et al. [43,44]. Table 1 presents the chemical composition in terms of the oxide of the materials used as binders that will be used in Section 2.2 to calculate the percentages of calcium oxides and silicon dioxide in each of the cement mixtures.

### 2.2. SCC Proportions and Fresh-State Properties

Nine SCCs, as well as one normal vibrated concrete (NVC), were used, as shown in Table 2. There are two reference compositions, C500 (500 kg/m^3^ of cement) and NVC (300 kg/m^3^ of cement), and eight compositions with the replacement of cement by fly ash (FA), with and without the use of metakaolin (M) and/or hydraulic lime (HL). The binder content varied between 500 and 400 kg/m^3^. Thus, for example, the mixture B500.FAMHL represents concrete with 500 kg/m^3^ of binder, using fly ash, metakaolin, and hydrated lime. Concerning NVC composition, it was produced to prove that its compressive strength is similar to that of low-cement concretes.

Immediately after the mixing process, a set of tests were carried out in the fresh state, namely slump flow (T500 and spreading), J-ring, V-funnel, and L-box with three bars, according to EN 206-9 [46]. The results are also shown in Table 2, and an in-depth discussion of these results can be found in the study by Anjos et al. [43].

The SCCs and NVC were placed in different moulds, depending on the test being carried out, and cured in water (20 ± 2 °C) for 28 or 90 days (until the planned tests were carried out).

Based on the volumetric fraction (*V_f_)* of each of the binders compared to the total binder present in the compositions (Table 2) and the respective oxide contents of each binder (Table 1), the percentages of calcium oxides and aluminium oxide in each of the mixtures were determined, considering Equations (1) and (2). The respective CaO and Al_2_O_3_ contents are presented in Table 3 and will be used in the carbonation, chloride-ion diffusion, and electrical resistivity analyses.

(1) $$\%CaO(composite)=V_{f}^{cim}*{CaO}_{cim}+V_{f}^{FA}*{CaO}_{FA}+V_{f}^{Mk}*{Cao}_{Mk\;}+V_{f}^{CH}*{CaO}_{CH}$$

(2) $$\%{Al}_{2}O_{3}\left(binder\right)=V_{f}^{cim}*{{Al}_{2}O_{3}}_{cim}+V_{f}^{FA}*{{Al}_{2}O_{3}}_{FA}+V_{f}^{Mk}*{{Al}_{2}O_{3}}_{Mk\;}+V_{f}^{CH}*{{Al}_{2}O_{3}}_{CH}$$

### 2.3. Test Programme

The mechanical performance of concretes produced was assessed considering the compression strength results. Since Anjos et al. [40] explored in detail the mechanical performance of these concretes, the present research only shows the 28- and 90-day results and focuses the discussion on durability performance. The tests carried out are described in Table 4.

## 3. Results and Discussion

### 3.1. Compressive Strength

Table 5 shows the results achieved for compressive strength at 28 and 90 days, as well as the percentage growth in strength between these ages.

As expected, a drop in compressive strength was observed when comparing the values obtained for SCC with partial replacement of cement with the reference concrete (C500). However, the values obtained for SCC with 500 kg/m^3^ of binder were very close and, in most cases, higher than those obtained for another reference concrete, NVC (300 kg/m^3^), at 28 and 90 days. SCC with 400 kg/m^3^ of binder presented values lower than those obtained by the NVC.

It was also observed that HL plays an important role in increasing resistance, with special importance at younger ages. All SCCs with HL showed greater compressive strength at 28 days than their respective references, SCC without HL. The same happened at 90 days.

The B500.FA and B500.FAHL contained a greater amount of cement (200 kg/m^3^) and FA (300 kg/m^3^) compared to the B500.FAM and B500.FAMHL samples (150 kg/m^3^ of cement and 250 kg/m^3^ of FA), which provided the first SCCs with greater resistance gains between 28 and 90 days when compared to the second ones. The same happens when B400.FA and B400.FAHL are compared to B400.FAM and B400.FAMHL. This increase in resistance is related to the greater availability of portlandite (free CH) for the reaction, as verified by Anjos et al. [44] in TG analysis at 28 days, and a greater pore filling effect caused by the FA that did not react.

### 3.2. Accelerated Carbonation Test Results

**Figure 2:**
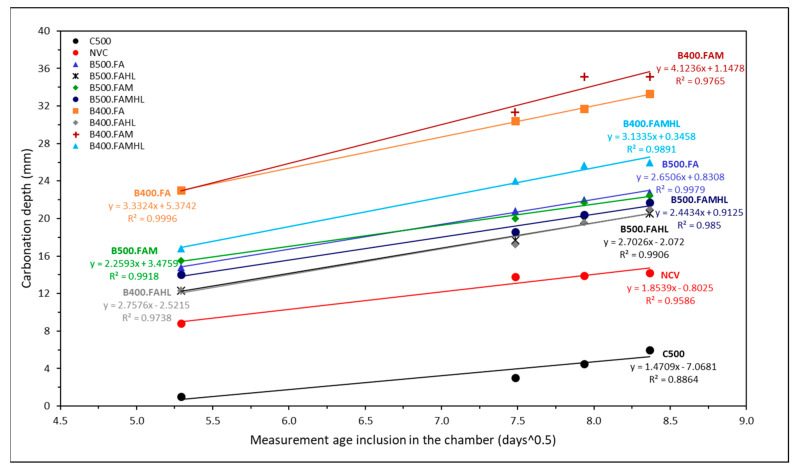
The relationship between carbonation depth versus the square root of the time spent in the carbonation chamber.

The accelerated carbonation test results for an exposure period of 28, 56, 63, and 70 days, after 42 days of curing, are presented in Figure 2. This figure shows the carbonation depth and the carbonation rate (k). “k” was approximated as the angular coefficient of the straight lines shown in Figure 2. However, it is known that the CO_2_ diffusion rate cannot be considered a constant, as it depends on several factors, including the degree of hydration, porosity, CO_2_ concentration, RH, quantities and types of microscopic phases, and type and content of mineral additions used, among other factors [51,52].

It is possible to verify that the carbonation depth is more pronounced in SCC with lower cement contents and without HL (B400FAM and B400FA). However, the reduction in carbonation depth with the addition of HL in the B400FA.HL and B400FAM.HL samples stands out. This reduction in the carbonation rate is 19.4% and 34.7% for the B400FA and B400FAM samples, respectively.

Concretes with only Portland cement (C500 and NCV) showed much lower carbonation depths than SCCs with partial replacement of cement with complementary cementitious materials (SCMs). This fact is related to the greater consumption of cement, and consequently, the higher content of free calcium hydroxide in the C500 and NCV samples compared to BF500, as shown by Anjos et al. [41] in a thermogravimetric analysis carried out on the pastes of these SCCs.

In concrete with mineral additions, the behaviour concerning carbonation is governed mainly by the low content of calcium hydroxide present in the hydrated matrix. This fact causes carbonation of other hydrated phases, as proven in the concretes B500FA, B500FAM, and B500FAM.HL, which do not present portlandite peaks in the analyses carried out on these compositions at 28 days, as demonstrated by Anjos et al. [40]. Increases in the carbonation of concrete with fly ash content are documented in the literature due to the reduction in portlandite content after the pozzolanic reaction and also the low content of calcium oxide (CaO) in the cement mixture [33,53,54,55].

The HL added to the SCC was important in reducing the carbonation depth in B500 and B400. When analysing the carbonation depth after 70 days of exposure in the chamber, there was a reduction in the depth of 8.9% and 3.4% in the B500FAHL and B500FAMHL samples, respectively, and 33.4% and 25.8% in the B400FAHL and B400FAMHL samples, respectively, compared to the SCC without HL. The carbonation rates of the B500FA and B500FAM samples and their respective SCC counterparts with lime, B500FA.HL and B500FAM.HL, are close to each other, which indicates the low efficiency of HL addition in these SCCs. However, the addition of HL is preponderant for B400.

Table 6 shows the results obtained for the carbonation depth at 28 and 70 days, followed by its growth percentage between these ages, as well as the comparative percentage between the carbonation depth of NVC and the other mixtures.

As expected, there was an increase in carbonation depth over time due to continuous exposure. Although the C500 mixture had a 500% increase in carbonation depth between the ages of 28 and 70 days, this high value is negligible, since it has a depth range of 6 mm, which is lower than the minimum cover for a non-aggressive environment [56]. The mixtures with additions obtained a greater carbonation depth compared to the NVC mixture, which is justified by the consumption of Ca(OH)_2_, which causes a reduction in the alkaline reserve and accelerates the propagation of the carbonation front. The carbonation depth observed was in the B400.FAM mixture (35.1 mm); nevertheless, the European standard EN 12390-12 [50] recommends a minimum cover of 40 mm for structural concrete present in an environment with a risk of carbonation-induced corrosion.

**Figure 3:**
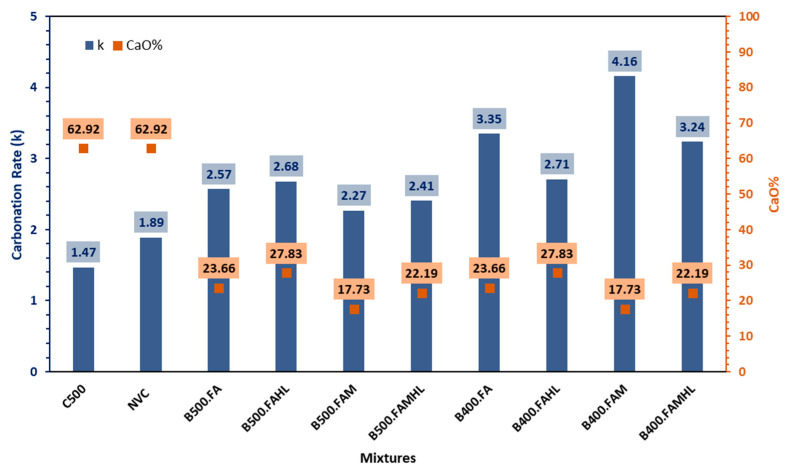
Relationship between CaO content and carbonation rate of mixtures.

Considering the important role of CaO in this context, Figure 3 shows a correlation between the k and the CaO content present in the concretes studied. It is observed that the lower the CaO content present in the SCC binders, the lower the CH_free_ content and consequently the greater the depth of carbonation.

Similarly to the results obtained in this study, Mohammed et al. [57] and Murtaza et al. [58] point out in their study that the greater depth of carbonation in the concrete samples may be associated with the higher percentage of SCM introduced. Zhong et al. [59] show that metakaolin and fly ash have a favourable influence on the carbonisation performance of concrete, and the carbonation resistance performance of concrete increases with the increase in metakaolin. Roberto da Silva et al. [60] found that adding HL to concrete with FA tends to reduce the carbonation coefficient of concrete at an early age. Diniz et al. [61] and Fonseca et al. [62] also proved in their studies that hydrated lime proved to be efficient in partially restoring the alkalinity of the SCC, providing lower depths of carbonation to SCC series that incorporated this addition.

### 3.3. Chloride Migration Coefficient (Non-Stationary Test) and the Relation with Void Index

Table 7 shows the chloride diffusion coefficients and the reduction in these coefficients in relation to the NVC mixture.

The use of mineral additions in concrete hinders the transport of chloride ions due to the refinement of its microstructure. In addition to the formation of new hydrated products, the aluminates present in cement and additions allow the production of Friedel’s salt when reacting with chloride ions. As chloride ions penetrate the concrete, the porosity of the concrete tends to decrease due to the formation of Friedel’s salt.

**Figure 4:**
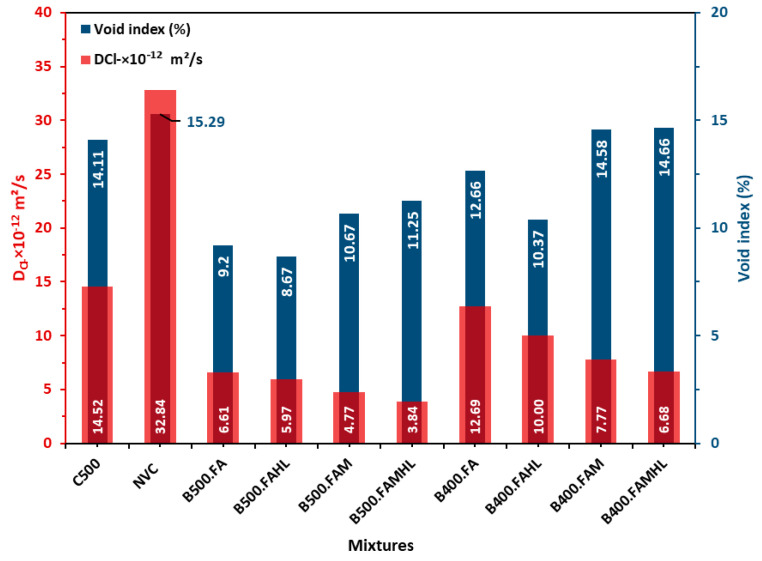
Diffusion coefficient, void index, and compressive strength at 90 days.

Considering the close relation between the chloride migration coefficient (D_Cl_) and the void index, these results are shown together in Figure 4 at 90 days (when the pozzolanic reactions are relevant). Regarding the chloride migration coefficient, it is evident that all SCCs with partial replacement of cement by SCM (binary, ternary, and quaternary) presented lower diffusion coefficients when compared to SCCs with only cement as a binder, even the one produced with a high cement content, C500 (500 kg/m^3^). Regarding the avoid index, the relation is similar, except for B400.FAM and B400.FAMHL, which have a higher avoid index than C500.

It is observed that the rate of permeable voids, cement consumption, and the content of mineral additions and hydrated lime in the SCC are preponderant in hindering the diffusion of chloride ions. Comparing the compositions with only cement as a binder, there is a reduction in the diffusion coefficient of 55.8% when the cement content is increased from 300 kg/m^3^ to 500 kg/m^3^, even with similar void rates of 15.29% and 14.11%, respectively, highlighting the importance of the cement content and the C_3_A content of the concrete.

However, it is noted that the most preponderant combination in the SCCs analysed was the combination of cement and mineral additions. This is because even in SCCs with low cement contents such as B500FA and B500FAMHL (cement consumption of 150 and 200 kg/m^3^), there is a reduction in the chloride-ion diffusion coefficient of 34.8% and 20.3%, respectively, concerning the reference C500 (500 kg/m^3^), and 79.9% and 88.3%, respectively, concerning the NVC (300 kg/m^3^). This fact is related to the ability of the hydrated products of the metakaolin pozzolanic reaction to fix the chlorides without leaving them free, especially the hydrated calcium silica aluminates (CaAl_2_Si_7_O_18_·2H_2_O) present in the B500 mixtures at 28 days and reported by Anjos et al. [43]. Peixoto et al. [63] obtained similar results, which showed that the addition of metakaolin to SCC with recycled aggregates was essential for reducing chloride diffusion.

It is also noteworthy that even SCCs with low compressive strengths, such as B400FA and B400FAMHL, with strengths of 28.0 MPa and 33.5 MPa, respectively, present lower diffusion coefficients than the reference C500 (62 MPa), confirming that the contents and types of mineral additions used are more preponderant in their behaviour against attack by chloride ions than cement consumption.

When analysing the additions of MK and FA in ternary SCC with low cement content (B500FAM and B400FAM), it appears that the joint use of these additions was relevant to reduce cement consumption to extreme limits such as 120 and 150 kg/m^3^, and yet they presented diffusion coefficients much lower than SCC with a consumption of 500 kg/m^3^. This fact is related to the increase in the alumina content in the SCC, which causes the formation of hydrated phases of the calcium silica aluminate type, resulting in a significant reduction in the diffusion coefficient of chloride ions. Other authors also identified the reduction in the diffusion coefficient using fly ash, metakaolin, and hydrated lime [30,31,64,65,66].

In addition, the ionic mobility in electrolytic solutions can be affected by the alkalinity of the pore solution [26,67,68]. Chloride-ion permeability results can be lowered significantly by reducing the concentration of alkali ions (Na^+^ and K^+^) and associated hydroxyl ions (OH^−^) [68,69].

The C500 and NCV reference concrete have higher chloride-ion diffusion coefficients. This is due to their greater alkalinity, in addition to the factors already discussed, since a thermogravimetric analysis carried out on their pastes, and previously presented by Anjos et al. [44], showed that the cement pastes of compositions C500 and NCV presented levels of free calcium hydroxide (CH_free_) of 8% and 15%, while the B500.FA, B500FAHL, and B500FAM presented CH_free_ contents of 1.2%, 7.2%, and 0.5%, respectively. This fact reflects the lower alkalinity of the BF500 and BF400 and, consequently, the lower diffusion coefficients presented by these samples compared to the reference concretes. The XRD analyses presented by Anjos et al. [40] for the B500FA, B500FAM, and B500FAMHL samples showed that there is no presence of portlandite, highlighting the importance of the pozzolanic reaction and the (OH^−^) content in the behaviour against attack by chloride ions in these SCCs with low cement content.

### 3.4. Electrical Resistivity

**Figure 5:**
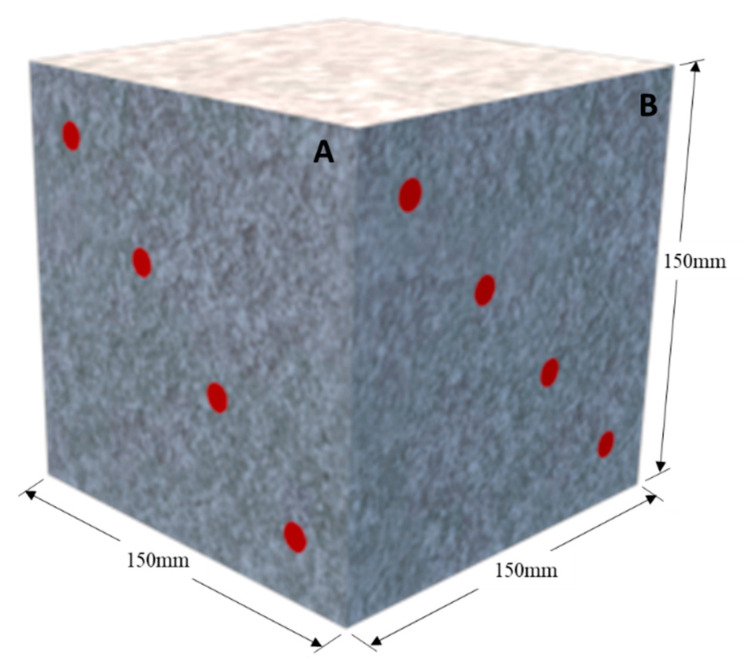
Electrical resistivity measurement points on the specimen faces.

**Figure 6:**
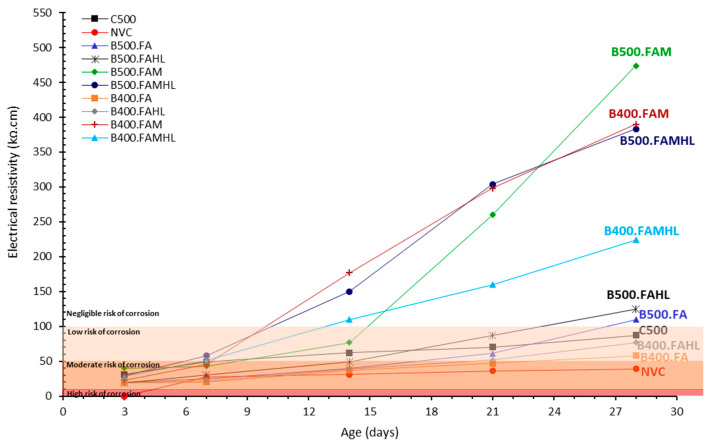
Electrical resistivity of the specimens for different ages.

The electrical resistivity measurements were carried out according to Figure 5. Four measurements were carried out on the diagonal of the specimen and perpendicular to each other on two distinct concrete surface faces (A and B). The average electrical resistivity value is shown in Figure 6. The figure is divided into four corrosion risk ranges, as suggested by Andrade and Alonso [70]: >100 kΩ·cm (negligible); 100–50 kΩ·cm (low); 50–10 kΩ·cm (moderate); and <10 kΩ·cm (high).

According to Figure 6, as expected, resistivity increases over time for all SCCs due to cement hydration and the progressive refinement of the microstructure, in agreement with other studies [63,71]. At three days, all SCCs showed a risk of corrosion between high and moderate. At 28 days, the risks are distributed between moderate and negligible according to their composition.

Electrical resistivity describes the ability of concrete to oppose the movement of ionic currents through its medium. Consequently, higher resistivity helps to inhibit the chances of corrosion in concrete; when resistivity increases, the rate of corrosion decreases [72]. In this sense, considering the 28 days of age, it can be seen that most of the SCCs with the partial replacement of cement show a risk of corrosion between low and negligible according to the ranges suggested by Andrade and Alonso [70]. All SCCs with 500 kg/m^3^ of binder and partial replacement of cement (B500.FA, B500.FAHL, B500.FAM, and B500.FAMHL) and two SCCs with 400 kg/m^3^ of binder (B400.FAM and B400.FAMHL) present a negligible risk of corrosion (>100 kΩ·cm). The B400.FA, B400.FAHL, and C500 present a low risk of corrosion (50 to 100 kΩ·cm). Only the NCV presents a moderate risk of corrosion at 28 days (10 to 50 kΩ·cm).

These results corroborate the diffusion data presented in Figure 4, in which all SCCs with 500 kg/m^3^ of binder and partial replacement of cement and the B400.FAM and B400.FAMHL samples present the lowest D_Cl_, between 3.84 and 7.77 (×10^−12^ m^2^/s). Then, B400.FA, B400.FAHL, and C500 present D_Cl_ values between 7.77 and 14.52 (×10^−12^ m^2^/s). The NVC presents the highest D_Cl_ value, 32.64 (×10^−12^ m^2^/s). Relating the results obtained in the two tests on chloride diffusion by migration and electrical resistivity, and based on the proposal by Andrade and Alonso [70] relating electrical resistivity and corrosion risk, it can be considered that a D_Cl_ value between 3.84 and 7.77 (×10^−12^ m^2^/s) indicates a negligible risk of corrosion and a D_Cl_ value between 7.77 and 14.52 (×10^−12^ m^2^/s) indicates a low risk of corrosion. This corrosion risk indicator related to the D_Cl_ may be used when resistivity tests are not available for concretes similar to the studied ones.

Some research relates the values of durability indicators related to chloride ions (diffusion coefficient and electrical resistivity) to the risk of corrosion of the reinforcement. According to Erdoǧdu et al. [73], the rate at which chloride diffuses from the surface into the steel can be related to the chloride-ion diffusion coefficient. When exposing a reinforced concrete structure to the marine environment for ten years, it has only a 4% chance of avoiding corrosion if D_Cl_ = 12 × 10^−12^ m^2^/s, whereas if D_Cl_ = 1.5 × 10^−12^ m^2^/s, the structure would remain almost intact.

The relationship between electrical resistivity, chloride diffusion coefficient, and Al_2_O_3_ content is close, as can be seen in Figure 5.

**Figure 7:**
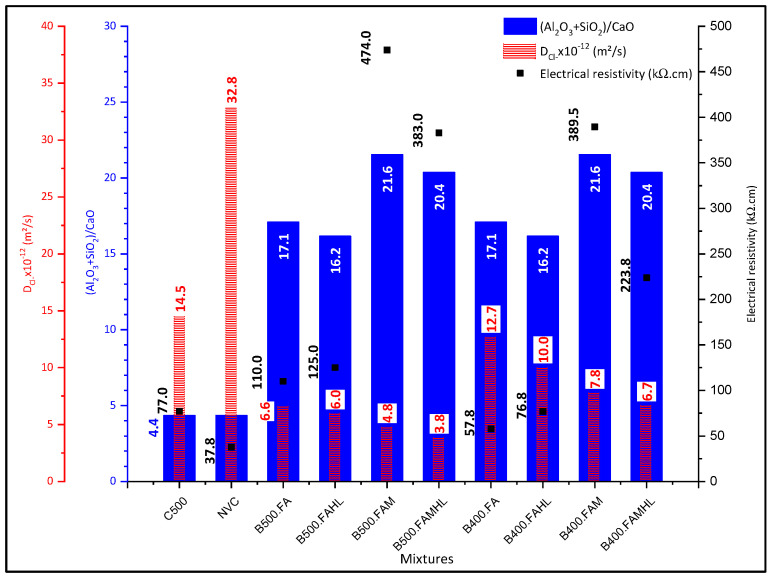
Relationship between Al_2_O_3_ content and electrical resistivity and chloride-ion diffusion at 28 days.

Concrete resistivity is also strongly affected by the type of pozzolan presented in the systems (Figure 6). The presence of metakaolin and fly ash and consequently the alumina content in the SCC resulted in low corrosion risks for the systems regardless of the amount of cement. The presence of HL did not result in a constant behaviour in all SCCs. When HL was used together with metakaolin, the resistivity decreased. When there was no metakaolin in the system, as is the case in binary mixtures B500.FA and B400.FA, resistivity increased, as shown in Figure 7. Thus, the use of mineral additions to obtain reduced-cement-content SCCs (150 and 200 kg/m^3^) instead of reference ones (300 and 500 kg/m^3^) did not decrease the resistivity of the concretes. On the contrary, all the binary, ternary, and quaternary blends, independent of binder content, showed bigger resistivity than the reference ones (C500 and NCV). In general, the 5% of hydrated lime addition was positive concerning resistivity.

## 4. Conclusions

Reducing the cement content in concrete is a huge challenge for the construction industry, motivated mainly by environmental issues. This reduction is more important for SCCs due to the need for high levels of cement in their traditional composition. To contribute to achieving this major objective, this research work studied self-compacting concrete with reduced cement content using SCMs. Following up on the study of mechanical properties developed by Anjos et al. [40], this work focused on the durability of these concretes in the face of the main mechanisms causing corrosion, carbonation, and penetration of chloride ions.

The novelty of this work lies in demonstrating that SCC with cement contents as low as 120–200 kg/m^3^ can achieve adequate durability properties, satisfying current needs for compressive strength and achieving satisfactory levels of chloride diffusion.

Regarding carbonation, the introduction of HL into the mixtures proved to be positive. The following main conclusions can be drawn from this research:

- The use of high levels of mineral additions strongly reduces the diffusion coefficients of chloride ions in concrete, with greater influence for mixtures with metakaolin at levels of 20% and cement consumption of 150 and 200 kg/m^3^.
- HL reduced the diffusion coefficients of chloride ions in all mixtures used due to the higher alkalinity of these mixtures compared to mixtures without HL.
- The use of MK and HL was decisive in reducing the diffusion coefficient of chloride ions.
- Accelerated carbonation was negatively affected by the high levels of mineral additions used. This behaviour is more severe when the additions are used together and/or with lower cement contents of the order of 120 to 160 kg/m^3^.
- The carbonation depth was reduced by up to 33% with the use of 5% hydrated lime in concrete with cement consumption of 120 and 160 kg/m^3^.
- The risk of corrosion measured through electrical resistivity is low or negligible in B500 and B400 SCCs (cement consumption of 120 to 200 kg/m^2^).
- There is a direct relationship between the higher Al_2_O_3_ content of the B500 and B400 mixtures and the lower chloride-ion diffusion coefficient. The presence of alumina in the mixtures resulted in low corrosion risks for the systems, regardless of the amount of cement present in the mixture.
- The combined use of MK, FA, and HL was relevant to achieve an important reduction in cement consumption, such as 120 and 150 kg/m^3^, and still presents durability indices compatible with SCC with a consumption of 500 kg/m^3^.

This study highlights that SCC with cement content as low as 120–150 kg/m^3^ can achieve durable performance when supplemented with SCMs. The use of metakaolin and hydrated lime significantly reduced chloride-ion diffusion and the risk of corrosion, with hydrated lime also proving effective in mitigating carbonation depths. These findings provide valuable insights into the development of more sustainable SCC, paving the way for its broader application in construction while effectively addressing critical durability challenges.

## Figures and Tables

**Table 1:** Chemical analysis of Portland cement, fly ash, metakaolin, and hydrated lime.

Materials	SiO_2_	Al_2_O_3_	Fe_2_O_3_	CaO	MgO	SO_3_	Na_2_O	K_2_O	LOI
PC (%)	19.92	4.36	3.51	62.92	1.83	2.86	nd *	nd	3.12
FA (%)	48.61	23.79	7.91	3.06	2.07	0.40	0.78	3.78	2.64
MK (%)	47.00	37.10	1.30	0.10	0.15	nd	0.20	2.00	11.75

* nd = not detected.

**Table 2:** The composition of the SCC and NVC (kg/m^3^) and fresh-state test results.

Materials (kg)									w/b (L/m^3^)	Slump Flow (mm)	T500 (s)	V-Test (s)	L-Box (H2/H1)	J-Ring (mm)
Concrete ID	C	FA	MK	HL	S	G	W	SP						
C500	500	-	-	-	870	880	200	13.0	1.3	625	1.67	4.6	0.75	
NVC	300	-	-	-	1053	867	180	7.8	1.8	500	4.20	-	-	
B500.FA	200	300	-	-	870	880	170	9.0	0.9	700	1.85	4.8	0.86	700
B500.FAHL	200	300	-	25	870	880	170	9.6	0.9	700	2.11	12.0	1.0	700
B500.FAM	150	250	100	-	870	880	170	9.6	0.9	670	3.15	13.9	0.92	615
B500.FAMHL	150	250	100	25	870	880	170	12.3	0.9	700	2.63	12.8	0.89	695
B400.FA	160	240	-	-	1034	916	140	12.4	0.9	750	1.83	5.85	0.94	705
B400.FAHL	160	240	-	20	1034	916	140	12.4	0.9	745	3.81	6.14	0.97	690
B400.FAM	120	200	80	-	1034	916	140	13.5	0.9	740	3.30	9.09	0.95	720
B400.FAMHL	120	200	80	20	1034	916	140	13.5	0.9	665	3.61	10.4	0.88	625

**Table 3:** The percentages of aluminium oxide and calcium oxides in the SCC and NVC.

Concrete ID	*V_f_* (Volumetric Fraction)				% Binder	
	C	FA	MK	HL	Al_2_O_3_	CaO
C500	1.00	0.00	0.00	0.00	4.36	62.92
NVC	1.00	0.00	0.00	0.00	4.36	62.92
B500.FA	0.34	0.66	0.00	0.00	17.10	23.66
B500.FAHL	0.32	0.62	0.00	0.06	16.17	27.83
B500.FAM	0.26	0.54	0.20	0.00	21.55	17.73
B500.FAMHL	0.24	0.51	0.19	0.06	20.38	22.19
B400.FA	0.34	0.66	0.00	0.00	17.10	23.66
B400.FAHL	0.32	0.62	0.00	0.06	16.17	27.83
B400.FAM	0.26	0.54	0.20	0.00	21.55	17.73
B400.FAMHL	0.24	0.51	0.19	0.06	20.38	22.19

**Table 4:** Tests performed.

Tests	Standard/Method	Specimen Dimensions (mm)	Testing Age (Days)	Conditions
Compressive strength	EN 12390-3 [47]	Cube—100 × 100 × 100	28 and 90	Test carried out after curing by immersion in water on the dates mentioned.
Void index	NBR 9778 [48]	Cube—150 × 150 × 150	90	Test carried out after curing by immersion in water on the date mentioned.
Chloride diffusion by migration	LNEC E-463 [49]	Cylinder—H200D100	90	Test pieces measuring H50D100 mm were tested.
Electrical resistivity	Wenner probe principle with four equidistant electrodes	Cube—150 × 150 × 150	3, 7, 14, 21, and 28	Measurement in the saturated state.
Accelerated carbonation	EN 12390-12 [50]	Cube—100 × 100 × 100	42 (entrance to the chamber)	Conditions: 4 ± 0.5% CO_2_, 20 ± 2 °C, and HR 55 ± 5%. Carbonation measurement: 28, 56, 63, and 70 days.

**Table 5:** Compressive strength results and strength increases from 28 to 90 days.

Concrete ID	Compressive Strength (MPa)		Strength Increases from 28 to 90 Days
	28 Days	90 Days	
C500	60.1	63.0	4.8%
NVC	32.1	34.9	8.6%
B500.FA	27.8	37.5	34.9%
B500.FAHL	40.9	58.3	42.5%
B500.FAM	32.6	38.3	17.5%
B500.FAMHL	40.0	46.5	16.3%
B400.FA	20.0	32.1	60.1%
B400.FAHL	28.4	40.7	43.1%
B400.FAM	23.0	28.3	21.9%
B400.FAMHL	27.5	33.5	22.0%

**Table 6:** Carbonation depth and percentage growth between ages.

Mixtures	Cement (kg/m^3^)	Carbonation Depth (mm) 28 Days	Carbonation Depth (mm) 70 Days	Carbonation Depth Strength Increases from 28 to 70 Days	Increased Depth of Carbonation Compared to NVC (28 Days)	Increased Depth of Carbonation Compared to NVC (70 Days)
C500	500	1.00	6.00	500.00%	−88.64%	−57.75%
NVC	300	8.80	14.20	61.36%	-	-
B500.FA	200	14.80	22.80	54.05%	68.18%	60.56%
B500.FAHL	200	12.30	20.50	66.67%	39.77%	44.37%
B500.FAM	150	15.50	22.40	44.52%	76.14%	57.75%
B500.FAMHL	150	14.00	21.70	55.00%	59.09%	52.82%
B400.FA	160	23.00	33.30	44.78%	161.36%	134.51%
B400.FAHL	160	12.30	21.00	70.73%	39.77%	47.89%
B400.FAM	120	23.00	35.10	52.61%	161.36%	147.18%
B400.FAMHL	120	16.80	26.00	54.76%	90.91%	83.10%

**Table 7:** Diffusion coefficient and percentage reduction.

Mixtures	Cement (kg/m^3^)	D_Cl_ (10^−12^ m^2^/s)	Reduced D_Cl_ Compared to NVC
C500	500	14.52	−56%
NVC	300	32.84	-
B500.FA	200	6.61	−80%
B500.FAHL	200	5.97	−82%
B500.FAM	150	4.77	−85%
B500.FAMHL	150	3.84	−88%
B400.FA	160	12.69	−61%
B400.FAHL	160	10.00	−70%
B400.FAM	120	7.77	−76%
B400.FAMHL	120	6.68	−80%

## Data Availability

The original contributions presented in this study are included in the article. Further inquiries can be directed to the corresponding author.
